# Polyphenols and Neurodegenerative Diseases: Potential Effects and Mechanisms of Neuroprotection

**DOI:** 10.3390/molecules28145415

**Published:** 2023-07-14

**Authors:** Raziel Alejandro Arias-Sánchez, Luz Torner, Bertha Fenton Navarro

**Affiliations:** 1 Laboratorio de Glicobiología y Farmacognosia, División de Estudios de Posgrado, Facultad de Ciencias Médicas y Biológicas “Dr. Ignacio Chávez”, Universidad Michoacana de San Nicolás de Hidalgo (UMSNH), Morelia 58020, Mexico; 1590741f@umich.mx; 2 Centro de Investigaciones Biomédicas de Michoacán, Instituto Mexicano del Seguro Social, Morelia 58341, Mexico; luz_torner@yahoo.com

**Keywords:** polyphenols, flavonoids, medicinal plants, antioxidants, neurodegenerative diseases

## Abstract

The increase in people’s longevity has, consequently, led to more brain involvement and neurodegenerative diseases, which can become complicated and lead to chronic degenerative diseases, thereby presenting greater public health problems. Medicinal plants have been used since ancient times and contain high concentrations of molecules, including polyphenols. It has been proven that polyphenols, which are present in various natural sources can provide curative effects against various diseases and brain disorders through neuroprotective effects. These neuroprotective effects are mainly attributed to their ability to cross the blood–brain barrier, eliminate reactive oxygen species, and cause the chelation of metal ions. Polyphenols increase the concentration of neurotrophic factors and bind directly to the membrane receptors of these neurotrophic factors, to modulate and activate the signaling cascades that allow the plasticity, survival, proliferation, and growth of neuronal cells, thereby allowing for better learning, memory, and cognition. Moreover, polyphenols have no serious adverse side effects resulting from their consumption.

## 1. Introduction

Due to the increase in life expectancy in humans and the increase in the number of older adults, there is predicted to be an increase of around 50% in people aged 60 to 80 years by the year 2030. Thus, one-third of the population will be over 65 years of age, and a quarter over 80 years of age. It is for these reasons that the group of patients suffering from neurodegenerative diseases (NDs) with severe neurological deficits and dementia is growing significantly, which is a critical public health problem. Faced with this enormous increase, investigations into the causes, risks, early diagnosis, and prevention of the disease, alongside its effective management are urgently needed for the affected patients [1,2,3,4].

Polyphenols are large phytochemicals that are found in natural sources of plant foods, fruits, cereals, spices, legumes, and beverages, such as tea, coffee, red wine, spices, and herbs, among other essential nutrients in the human diet [4,5,6,7,8]. Polyphenols are abundantly ingested in the human diet, where more than 1 mg of polyphenol content per serving can be consumed [8], and up to 1 g of median total polyphenol intake per day [9,10]. In addition, they contribute to the bitterness, color, flavor, odor, astringency, and oxidative stability of food [11,12]. Furthermore, plant polyphenols protect the plant from reactive oxygen species (ROS), ultraviolet (UV) radiation, pathogens, parasites, and plant predators [13].

The search for natural strategies to enhance healthy aging is promoting the extensive study of plant polyphenols to prevent deterioration and age-associated diseases, including NDs. In vitro, cell-based, animal, and human studies have attempted to decipher the mechanisms behind the neuroprotection of dietary polyphenols. Therefore, this review will address the beneficial action of these compounds in the brain and highlight their beneficial effects in various neurodegenerative pathologies.

### 1.1. Neurodegenerative Diseases (NDs)

NDs are a group of disorders of the central nervous system (CNS), which are characterized by populations of neurons that progressively lose their functions and connections [3], resulting in sensory and motor deficits and cognitive impairment [13]. NDs are broadly classified through their clinical symptoms, the most common being extrapyramidal and pyramidal movement disorders, and cognitive or behavioral disorders [3].

In NDs, the accumulation of ubiquitin-proteasomal systems (amyloid deposits) and neurofibrillary tangles (or Lewy bodies) generates oxidative stress (ROS), excitotoxicity, neuronal and synaptic dysfunction, impairment of protein degradation systems, endoplasmic reticulum (ER) stress, mitochondrial dysfunction, DNA damage, inflammation, and re-entry into the cell cycle, eventually causing apoptosis [3,14,15,16,17,18,19,20].

The most common NDs include Alzheimer’s disease (AD), Parkinson’s disease (PD), multiple sclerosis (MS), epilepsy, Creutzfeldt–Jakob disease, and Huntington’s disease (HD). All diseases have a progressive course with clinical and biochemical changes that affect the CNS [3,20,21].

The brain captures 20% of the total oxygen consumed with a high proportion of easily peroxidizable polyunsaturated fatty acids [22,23], generating large amounts of ROS in the brain. A change in the balance between the generation of ROS and the elimination or detoxification of these species is denoted as “oxidative stress”, which is a condition associated with chronic diseases causing cell death [14]. These events are more common in the high and sustained production of ROS and reduced levels of antioxidant defenses, as occurs in various pathologies and during the process of normal aging [13,14,23].

NDs and some chronic degenerative and cardiovascular diseases increase the possibility of causing damage to the CNS, causing regional degeneration of the brain and cognitive and behavioral disorders. The link between diabetes, hypertension, cardiovascular disease, and degenerative brain disease is well established, as these diseases can cause cognitive impairment, regional brain degeneration, memory impairment, diabetic neuropathy, brain loss, and memory impairment, and may eventually cause AD, and an alteration to the blood–brain barrier (BBB), thereby promoting neuroinflammation and exacerbation of amyloid pathologies among other brain affectations [18,24,25,26,27,28,29,30].

### 1.2. Medicinal Plants and Secondary Metabolites

Medicinal plants are a therapeutic alternative with various pharmacological properties due to their different chemical components, which can act individually or in synergy. In addition, they are well known for having fewer or no side effects [31,32,33].

Plants produce essential compounds for survival, growth, development, and reproduction, such as sugars, proteins, amino acids, and secondary or non-essential products [33,34,35]. These secondary metabolites are essential to defend plants against biotic or abiotic stress. However, they also attract pollinators and serve as signals or regulators in plant-environment interactions [35,36,37].

Plant secondary metabolites have been widely studied for health maintenance and the prevention, diagnosis, amelioration, or treatment of physical and mental illnesses [32,38]. In the pharmaceutical industry, they are particularly interesting since they are used as drugs or in the development of new drugs [32]. In addition, they are also considered as food additives for therapeutic, aromatic, and culinary purposes, as cosmetics, chemicals, and, more recently, as nutraceuticals, which has exponentially increased their commercial importance and value [11,31,39].

Secondary metabolites are structurally and chemically diverse, meaning they are classified according to their structural and biosynthetic pathway similarities: fatty acids and polyketides (from the acetate pathway), phenylpropanoids, polyphenols, and aromatic amino acids (from the shikimate pathway), terpenoids and steroids (from the mevalonate pathway), and nitrogen-containing compounds (alkaloids) [36,37,38,40,41,42].

## 2. Polyphenol Synthesis and Classification

More than 8000 types of polyphenols with various bioactivities are classified into subclasses, although the inclusion criteria can vary. These can be based on the source where they are found, the chemical structure, or their biological function [7,43].

The synthesis of polyphenols in plants requires the process of photosynthesis and the ubiquitous pathway in shikimate plants [40]. The shikimate pathway produces three essential amino acids, L-tryptophan, L-phenylalanine, and L-tyrosine, which the plant uses to synthesize crucial proteins for growth, the generation of pigments, hormones, and complex aromatic compounds (alkaloids, phenylpropanoids, polyphenols), and the synthesis of cell wall components [43,44,45]. During plant photosynthesis, the pentose phosphate pathways form primary metabolites, such as phosphoenolpyruvate (PEP) and D erythrose-4-phosphate (E-4-P). Shikimate is formed through the enzyme 3-deoxy-D-arabino-heptulosonato-7-phosphate synthase (DAHP), which in turn, is formed by dephosphorylation reactions that create chorismate—an initiator in the synthesis of aromatic amino acids—such as tryptophan, phenylalanine, and tyrosine, which form phenolic compounds [41,45,46].

**Figure 1:**
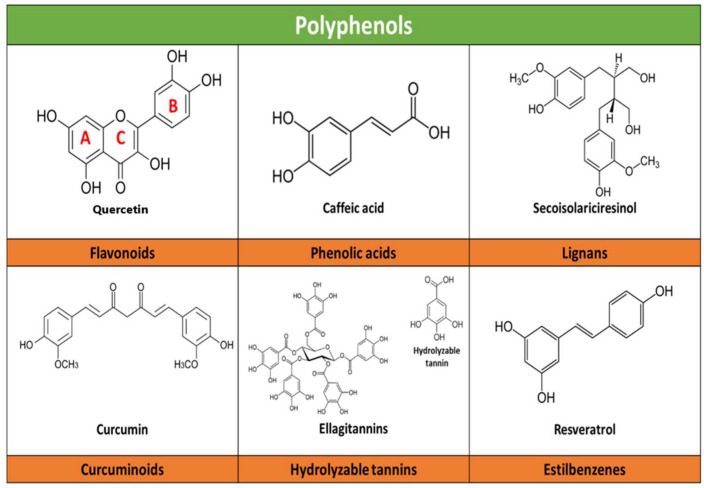
Schematic illustration of the main chemical structures belonging to the polyphenol subclasses and examples of each. An example of the main compounds of each polyphenol subclass and their chemical structure is presented. A and B refer to phenyl rings, C refers to a pyran ring (heterocyclic).

Structurally, they are characterized by one or more aromatic rings attached to one or more hydroxyl (HO) groups. Most polyphenols are characterized by the presence of two aromatic benzene rings (A and B rings), which are linked by a pyranic ring (C ring); depending on the degree of oxidation of the C ring, can be classified into six subclasses: flavonols, flavanones, isoflavones, flavanols, anthocyanins, and flavones. In addition, phenolic acids, lignans, curcuminoids, and stilbenes exist (Figure 1). The various functional groups or the degree of oxidation of polyphenols allow them to create non-covalent bonds (hydrophobic and hydrogen) and covalent bonds with proteins and carbohydrates [6,7,10,21,47,48,49]. Flavonoids constitute the largest class of polyphenols, including almost half of all phenolic compounds identified to date [7,10,48,49,50,51].

The distribution of phenols in plants at the tissue, cellular, and subcellular levels is not homogeneous since insoluble phenols are found in cell walls, while soluble phenols are inside the vacuoles of plant cells. The outer layers of plants have higher levels of phenols than those in the inner layers. Various factors affect the content of polyphenols in plants, including the degree of maturity at harvest, environmental factors, the part of the plant used, processing, and storage [7,11,12,52].

### 2.1. Digestion, Absorption and Distribution

In foods, polyphenols are identified as esters, glycosides, aglycones, or polymers, which have low bioavailability (total drug concentration in the target organs) or cannot be absorbed within the body. Due to this low bioavailability, polyphenols need to be hydrolyzed to be absorbed. The factors involved in their absorption are the chemical structure, the degree of glycosylation, and the percentage of polyphenols received by the colon and the enterohepatic circulation [7,9,10,42,43,53]. In addition, the type of microbiota found in each organism plays an essential role in the metabolism and absorption of polyphenols [7,10,54].

The digestion of polyphenols begins from the chewing process in the mouth and with the secretion of saliva and microbiota, which possess β-glucosidase activity, thereby reducing polyphenols to simpler forms [42,54,55]. However, polyphenols cannot be absorbed in the stomach since they resist gastric secretions (except for some, such as quercetin and anthocyanins). The polyphenols that reach the colon undergo several reactions through the microbiota and intestinal enzymes, such as oxidation, reduction, hydrolysis, methylation, sulfation, and glucuronidation, to become aglycones. This favors their polarity and their hydrophilic property, which in turn, facilitates their absorption, increases their physiological activity, and reduces possible toxic effects [40,49,53,54,55,56]. The presence or absence of certain bacteria in the microbiota will cause various bio-transformations in such a way that the beneficial effects of polyphenols depend on the type of polyphenols consumed and the composition of the microbiota [13,55,56]. Due to these modifications, the polyphenol forms in plasma differ from those found in their natural sources [40]. Once the biomodifications of the polyphenols are made, they can be absorbed by enterocytes and colonocytes [13]. The conjugated polyphenols enter the circulation for distribution to the organs and subsequent excretion through bile, feces, or urine. Polyphenols circulate in the blood bound to proteins, such as albumin, which is the main binding protein. Thus, polyphenols are metabolized and transported to target tissues (lung, pancreatic, brain, cardiac, and splenic). The reuptake of polyphenols that reach the intestine through the portal vein, which are ready for elimination, sends them to the liver for transport back to the target organs [40,49,53,57].

It is essential to emphasize that the chemical structure of the polyphenols and not their concentration determines the speed, the degree of absorption, and the nature of the metabolites that circulate in the plasma. The most common polyphenols in our diet are not necessarily those that show the highest concentration of active metabolites in target tissues; consequently, the biological properties of polyphenols differ significantly from one polyphenol to another [12,40,51,53]. By consuming foods or natural products with a high and heterogeneous content of polyphenols (polyphenol cocktail), much more efficient results can be obtained in a shorter time, unlike isolated natural or pharmaceutical chemical compounds.

### 2.2. Safety of Polyphenol Consumption

Human research has reported that consuming natural polyphenols and pharmaceutical products that provide alternative polyphenols to those typically found in the diet is safe and tolerable in the short, medium, and long term [53,56,58]. However, if consumed in excess, they can reduce the bioavailability of trace elements, vitamins, or folic acid and cause alterations in thyroid function [52,58]. Other rare, mild, and transient side effects include minor gastrointestinal problems, headaches, dizziness, and rashes [53].

There is a lack of information on how they work and on the side effects from consuming various products containing polyphenols may have on their interactions with other polyphenols and other molecules, whether food supplements, nutraceuticals, drugs, or food [52,53]. Such is the case in the polyphenols found in grapefruit juice, such as apigenin, naringenin, nobiletin, and hesperetin, which potently inhibit detoxifying enzymes, members of the CYP family, responsible for the metabolism of various prescription drugs [47,59,60].

A constant limitation in using polyphenols from natural products is their bioavailability; thus, plant products exerting neuroprotective actions are positively related to the amount consumed. Pharmaceutical strategies, such as the encapsulation of plant extracts using biodegradable materials, lipid nanocapsules, nanoparticles, exosomes, nanocomposites, and emulsified or gel-formulated formulations, are currently in progress to promote the enhancement of phenolic bioavailability, BBB permeability, and its therapeutic efficacy [4,53]. The self-nanoemulsifying drug delivery system improved the pharmacological activity and showed neuroprotective effects [61].

### 2.3. Polyphenols as Antioxidants

ROS are produced by the metabolism of physiological processes; when they reach specific concentrations, they control the redox homeostasis of the cells, causing an imbalance between the production of ROS and the antioxidant cell defense capacity [62]. This causes protein and lipid oxidation, DNA oxidation, and glycoxidation, which affects cell structure and functional integrity, resulting in cell damage and necrotic or apoptotic cell death [13]. The excessive production of ROS in neuronal cells is mainly due to the activity of excitatory amino acids and neurotransmitters [63].

There is an increased interest in bioactive compounds produced by plants due to their beneficial effects on human health, such as their antioxidant activity. Antioxidant compounds, when isolated and identified, can be used to develop, and formulate various products related to food, cosmetology, and pharmaceuticals. Antioxidants are essential to prevent oxidative damage in living cells and oxidative spoilage in food. These compounds can delay oxidative degradation reactions by forming complexes with pro-oxidant metals- They can reduce the oxidation of cell structures by eliminating free radicals or inhibiting their production, thus, delaying or blocking cell damage [4,64].

Antioxidants, such as phenolic compounds, particularly those whose hydroxyl groups are in the ortho or para position, play a role in redox reactions, acting as reducing agents and hydrogen donors [53]. An important point to consider is the number of hydroxyl groups within each phenolic compound; the higher the number, the greater its antioxidant efficacy may be [4].

**Figure 2:**
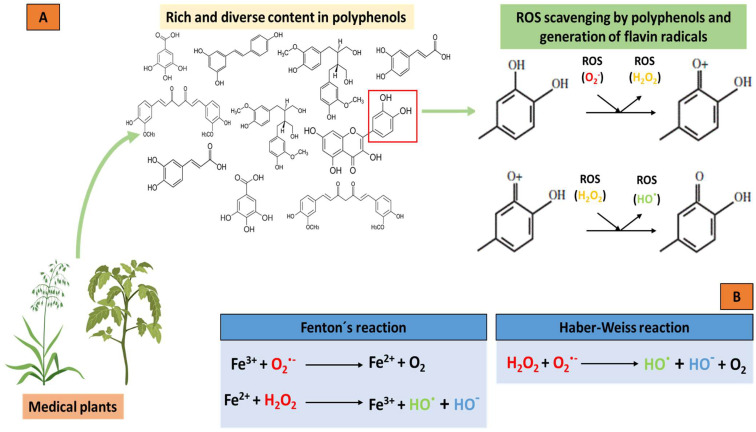
Antioxidant potential of polyphenols from medicinal plants and the chelation of metal ions by the Fenton and Haber–Weiss reactions. (**A**) Donation of protons from the polyphenols to the radicals, generating less reactive molecules and the subsequent formation of the flavin radical due to the capture of free radicals in the polyphenols. (**B**) Chelation of metal ions and the elimination of ROS by Fenton and Haber–Weiss reactions. Oxygen (O_2_); superoxide radical (O_2_·^−^); hydrogen peroxide (H_2_O_2_); hydroxyl radical (HO·); hydroxyl ion (HO^−^).

Thus, polyphenols interfere with the oxidation of biomolecules by rapidly donating protons to radicals or by reacting with radicals to form compounds that prevent them from reacting with other molecules. Furthermore, polyphenols can interact with receptors or enzymes in signal translation, thus, modifying cellular oxidation and promoting an antioxidant condition. Flavonoids act as buffers and capture free radicals to generate the flavinic radical, which is much less reactive since the unpaired electrons are more delocalized (Figure 2A). Furthermore, some flavonoids can chelate transition metal ions, such as iron or copper, thereby preventing the formation of the ROS produced by the Fenton and Haber–Weiss reactions (Figure 2B). Polyphenols can also interfere with cell detoxification systems, such as superoxide dismutase, catalase, or glutathione peroxidase, thereby increasing the activity of these enzymes. Therefore, polyphenols are known as excellent antioxidant molecules.

There is a direct positive correlation between the antioxidant capacity of polyphenols and the number of hydroxyl groups present in the phenolic structure. Furthermore, the antioxidant effect is reduced only to eliminating and blocking the effects of free radicals. Therefore, the strategies for the antioxidant activity of polyphenols go beyond the neutralization of free radicals and include other activities, such as iron chelation, regulation of signaling pathways, and intervention in the cell cycle of cells from different organs [5,14,23,48,65,66,67].

## 3. Polyphenols in the Nervous System and Their Neuroprotective Effects

Polyphenols are believed to be present in low concentrations in the brain (1 nmol/g of tissue), making them sufficient to affect neuronal pathways since they can cross the BBB by diffusion, either as aglycones or as their conjugation products. The ability of flavonoids to permeate the BBB depends not only on their lipophilicity but also on their conjugation capacity; metabolites that are conjugated by methylation in the small intestine and the liver, being more lipophilic (less polar), can permeate the BBB faster than their aglycones of origin. Less lipophilic polyphenols cross the BBB when they bind to specific ATP-dependent transporters. Polyphenols can directly interact with neurotransmitters in the signaling cascades of several kinases, such as MAPK, PI3K, and PKB [13,14,21,40,54,67,68,69,70].

In recent decades, polyphenols have been exhaustively studied for their prevention and possible treatment of age-related NDs. Consequently, a wide range of polyphenols are known to influence neuronal function [4,6,47,71] and provide pleiotropic effects in neuronal cells [21]. Diets rich in polyphenols have been shown to provide benefits for maintaining cognitive functions due to the survival, differentiation, and improvement of neuronal function and regeneration [6,53,72,73,74,75]. Furthermore, polyphenols stop the progression of NDs by positively affecting memory, learning, and cognition [75]. The neuroprotective effect of flavonoids in AD is related to the mediation of glycogen synthase kinase 3β (GSK3β) and cyclin-dependent kinase 5 (CDK5) [76].

The latter is from a direct neuroprotective approach. However, polyphenols also provide indirect neuroprotective effects by modulating the composition of the intestinal microbiota and the metabolites produced, among other mechanisms. Both approaches modify the production of neurotransmitters and neuropeptides, to influence brain functions [13,55].

In the treatment of ND, there are MAO-A inhibitors for the treatment of mental disorders, such as depression and anxiety, and MAO-B inhibitors for the treatment of neurodegenerative disorders, such as Alzheimer’s and Parkinson’s diseases. Flavonoids can inhibit monoamine oxidase-A and monoamine oxidase-B [77].

Polyphenols improve the regulation of neuronal survival, acting through different points of signaling pathways; this may be a promising approach for treating CNS diseases [28]. Brain-derived neurotrophic factor (BDNF) and nerve growth factor (NGF), members of the neurotrophin family, are associated with the development and regeneration of neurons (neurogenesis) and long-term potentiation in the hippocampus; they also induce structural changes in synapses and survival and resistance to neuronal damage, all of which are widely attributed as a critical objective in neuroprotection through the presence of polyphenols [75,78,79].

The CNS and peripheral tissues mainly produce neurotrophins and are closely related among humans, rats, and mice [78,80]. There is a positive correlation between cognitive performance and the concentration of BDNF in the brain. In contrast, decreased BDNF production has been identified as a possible pathogenic factor in brain diseases and disorders in animals and humans [75,79,81].

**Figure 3:**
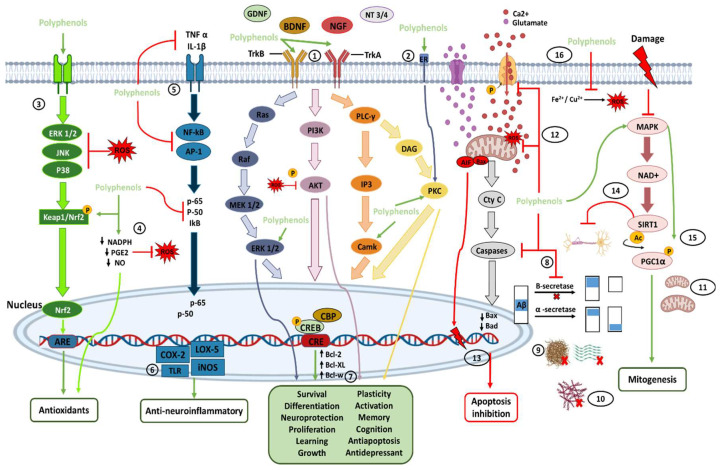
Direct (path-ways 1–11) and indirect (path-ways 12–16) signaling pathways of polyphenols involved in brain neuroprotection. Polyphenols provide antioxidant effects mainly through the direct and indirect elimination of ROS and through the Nrf2 pathway. Polyphenols also have anti-neuroinflammatory effects by inhibiting and modulating cytosines and proinflammatory transcriptional factors. In addition, by binding directly with neurotrophic factors and their receptors, polyphenols can activate pathways of neuronal survival, growth, proliferation, and neuroprotection, among others. Polyphenols can also control the inhibition of apoptosis and increase mitogenesis by inhibiting proapoptotic molecules and activating the MAPK pathway. In addition, polyphenols provide several indirect pathways of neuroprotection, each of which is described in more detail in the text. The numbers enclosed within the circles indicate the number of the path-way to which they refer in the text.

The transmembrane protein tropomyosin kinase B (TrkB) receptor is a specific receptor for BDNF, while TrkA is the receptor for NGF, which is widely expressed in the mammalian brain. BDNF/NGF and TrkB/TrkA, and glial cell line-derived neurotrophic factor (GDNF) are essential for adult synaptic plasticity, memory formation, neurite outgrowth, neurotrophic activities, and the activation of neuroprotective pathways. Activation of TrkB is faster (approximately 2 min), and its deactivation occurs within 30 min. Both BDNF and polyphenols, when stimulating TrkB, activate three important downstream intracellular signaling cascades, including the PI3K/Akt, phospholipase C-γ (PLC-γ), and MAPK/ERK pathways (path-way 1 in Figure 3)

These signaling cascades, ultimately, lead to the phosphorylation of the cAMP response element-binding protein (CREB) and regulate transcription in neurons [53,67,78,81,82]. CREB is a cellular transcription factor in higher eukaryotes and is relatively abundant in the brain, particularly in neurons. The number of surviving neurons is closely related to the concentration of phosphorylated CREB (pCREB), which plays an essential role in learning and memory in the brain [27]. In the hippocampus, it has been shown that activation of phosphatidylinositol-3-kinase (PI3K) and its downstream effector on Akt could upregulate CREB phosphorylation, and prevent neuronal death [80,81,82,83]. BDNF/TrkB and NGF/TrkA signaling are involved in neuronal survival, memory formation, antidepressant-like effects, neural plasticity, and stress resistance. The ERK (extracellular signal-regulated kinase) pathway, a part of the mitogen-activated protein kinases (MAPKs), has been involved in various physiological functions of neurons, including proliferation, differentiation, and survival (by the induction of survival genes and the inhibition of proapoptotic proteins). ERK1/2 is activated after the phosphorylation of threonine and tyrosine residues, which changes its localization and phosphorylation of different target molecules. It has been proposed that CREB, a downstream regulator of the ERK cascade, is involved in neuronal proliferation (neurogenesis), neuroplasticity, emotion, and cognition.

Ca^2+^/calmodulin-dependent protein kinases (CaMKs) are a family of serine/threonine protein kinases (CaMKI, CaMKII, and CaMKIV). CaMKII is abundantly expressed at postsynaptic sites, and its activation contributes to synaptic protein phosphorylation. CaMKIV is found primarily in neuronal nuclei and is crucial for long-term memory, in the brain, by activating CREB, which stimulates the transcription of target genes by binding to the DNA cAMP response element (CRE) region. In addition, the CREB pathway positively affects cognitive health, neuronal survival, neurogenesis, synaptic plasticity, and general neuronal activation. In this regard, polyphenols can bind to the estrogen receptor (ER) and activate neurotrophic effects through protein kinase C (PKC) pathways (path-way 2 in Figure 3) [13,23,27,70,78,82,83,84,85].

On the other hand, in neuroinflammatory diseases, such as MS, the administration of polyphenols increases the production of BDNF, which may show neuroprotective activity due to its immunomodulatory action. Therefore, polyphenols can be used as a therapeutic strategy in detecting and preventing inflammatory neurological disorders and, generally, enhance neuroprotection [77,78]. Likewise, there are reports that intestinal microbes can directly affect the release of BDNF, dopamine, serotonin, GABA, catecholamines, and histamine in the brain. Thus, having a healthy microbiota can increase these molecules and lead to neuroprotective effects. [23].

Another neuroprotective effect of polyphenols in the brain is the elimination of ROS. Polyphenols induce an antioxidant effect by activating protein kinases that signal molecular pathways, such as Keap1/Nrf-2/ARE, the main protection pathways against endogenous and exogenous ROS [84,85,86]. Polyphenols interact and activate receptor kinases ERK1/2, JNK, and p38 protein, to disrupt the Keap1/Nrf2 complex and allow the translocation of nuclear factor 2 (Nrf2)-related transcription factor to the nucleus, where it binds to receptors rich in adenylate and uridylate (ARE), and promotes the expression of antioxidant proteins and enzymes (GSH, GST, GPX, SOD, and CAT) (path-way 3 in Figure 3). Polyphenols also have antioxidant properties through the inhibition of NO and PGE2 production, thereby decreasing the activation of NADPH oxidase, and preventing the formation of ROS in the brain (path-way 4 in Figure 3) [4,13,27,40,51,53,65,67,82].

Polyphenols have anti-neuroinflammatory activities by blocking the release of cytokines (IL-1β, TNF-α), inhibiting Nf-kB expression, and activating protein 1 (AP-1). In addition, the MAP pathway, mediated by TNF-α, inhibits the synthesis of proinflammatory cytokines, such as p-65, p-50, and IkB (path-way 5 in Figure 3) [13,65,87]. In turn, the inhibition of Nf-kB downregulates microglial activation and the subsequent release of proinflammatory factors, in addition to the production of the enzymes COX-2, LOX-5, inducible nitric oxide synthase (iNOS), and nitric oxide (NO). Polyphenols also attenuate the phosphorylation and overactivation of MAPKs, such as IkB and p38 MAPK, to protect against dopaminergic neuronal death. Finally, the overexpression of TL receptors, such as TLR2, TLR4, TLR9, and Myd88, is avoided (path-way 6 in Figure 3) [4,48,51].

As described before, polyphenols can increase the synthesis of neurotrophic factors, such as BDNF, NGF, neurotrophin 3 (NT3), and neurotrophin 4 (NT4), as well as increase the ability to bind directly to Trk receptors and regulate transcription, translation, proliferation, growth, and survival through pathways such as PI3K/AKT, PCK-ERK1/2, Akt-ERK1/2, and MAPK, STAT3 activating CREB. This increases and adjusts the expression of B-cell lymphoma-2 (Bcl-2), Bcl-2-associated protein X (BAX), Bcl-2-like protein 2 (Bcl-w), and B-cell lymphoma extra-large (Bcl-XL), which induces antiapoptotic effects on the neurons and improves neuropathies. All these effects are possible because polyphenols can cross the BBB to access brain lesions [13,40,43,51,65,67,82]. In addition, many polyphenolic compounds present antidepressant effects (side effects of neurodegenerative damage) associated with elevated BDNF levels (path-way 7 in Figure 3) [49,50,82].

Polyphenols also prevent protein deposition through several mechanisms, such as antioxidant properties, the degradation of amyloid precursor proteins by α/β-secretase into non-amyloidogenic proteins, through the cleavage of APP by α-secretase (path-way 8 in Figure 3), by direct binding to the protein to avoid its fibrillation (path-way 9 in Figure 3), or even the prevention of tau aggregation and hyperphosphorylation (path-way 10 in Figure 3), all of which protects the CNS from the accumulation of Aβ plaques [4,48]. Furthermore, phenolic compounds can interact with hydrophobic Aβ and αS sequences by disrupting the β-sheet motifs, preventing tau hyperphosphorylation, and making the proteins smaller and non-toxic. This also helps to reduce amyloidogenic effects, the loss of dopaminergic neurons, and apoptosis [4,50]. In addition, the activation of MAPK by polyphenols helps to upregulate the mitochondrial biogenesis necessary for dopaminergic neuronal survival (path-way 11 in Figure 3). It has also been found that polyphenols increase dopamine, serotonin, and norepinephrine, in the hippocampus and frontal cortex, which are regions of the brain that play a key role in the pathophysiology of mood disorders; thus, reducing depressive and anxious behavior [23,50].

## 4. Other Neuroprotection Approaches (Indirect Mechanisms)

Excitotoxicity is mediated by elevated intracellular Ca^2+^ and nitric oxide-mediated oxidative stress, resulting in DNA damage, peroxisome proliferative activated receptor (PPAR) activation, NAD^+^ depletion, and cell death [50]. In addition, excessive activation of glutamate receptors causes a massive influx of calcium and alterations in the membrane potential of the mitochondria, increasing the production of ROS, and triggering apoptosis in different brain diseases due to the increase in BAX, cytochrome C, and caspase activation. This excitotoxicity also affects the mitochondrial intermembrane space (path-way 12 in Figure 3) [4,14].

Excitotoxic events also induce AKT and ERK activation, which are essential for neuronal death due to increased calcium, protein kinase A, diacylglycerol, and cAMP levels. In addition, the increase in Ca^2+^ activates the transcription factor NF-kB in neurons [4]. On the other hand, an apoptosis-inducing factor (AIF) is a protein released from mitochondria into the cytosol. It translocates to the nucleus to initiate DNA fragmentation, chromatin condensation, and caspase-independent cell death. In addition, AIF is cleaved and released into the cytosol by the presence of Ca^2+^ and participates in energy loss, free radical generation, and death processes. In turn, Bax is a protein that usually resides in the cytosol. However, during apoptotic events, Bax moves from the cytosol to the mitochondrial outer membrane. Once there, this protein releases apoptogenic factors into the cytosol from the mitochondrial inner membrane space, including cytochrome C and AIF (path-way 13 in Figure 3). Polyphenols exert neuroprotective effects, inhibiting cell death and attenuating the depolarization of the mitochondrial membrane induced by the activation of glutamate receptors, to reduce the entry of Ca^2+^. Furthermore, polyphenols following excitotoxic stimuli in the neurons prevent the overexpression of Bax in the cytosol and block its translocation to mitochondria, thereby disrupting its translocation caspase- and AIF-dependent apoptosis and reducing mitochondria-dependent apoptotic neuronal death. At an early stage, by inhibiting the ERK, ROS, Bax, AIF, and mTOR pathways to cause autophagy and activate caspase-3, polyphenols can prevent or promote cell death as needed [4,14,48,65,67,70].

Polyphenols have been shown to inhibit acetylcholinesterase and neutralize the abnormal folding of tau proteins, thereby preventing AD and dementia [69]. Polyphenols also limit demyelination through SIRT1 activation and attenuate neuronal damage in MS (path-way 14 in Figure 3) [66,88]. In addition, several reports have shown that the frequent consumption of polyphenols reduces the risk and severity of stroke by downregulating matrix metalloproteinases (MMP) and controlling the deregulation of calcium and lipid peroxidation in neurons [14,88]. Polyphenols may also function as therapeutic molecules in HD by SIRT1 activation of the PPARγ and coactivator 1 alpha (PPARGC1A) signaling pathway (path-way 15 in Figure 3) [67,88].

Metal ions such as iron and copper have an essential role in the generation of ROS. Moreover, the accumulation of metals in the brain contributes to pathologies such as AD, MS, PD, and HD [87,88,89]. Thus, polyphenols are potent metal chelators and extend neuroprotection against the oxidative stress caused by iron and copper, as well as neurotoxicity due to metal chelation, the modulation of signal transduction, and inflammation, which also prevents damage to neuronal DNA (path-way 16 in Figure 3) [13,54,65,67,70].

Intestinal microbiota improves the bioavailability of polyphenols; thus, it can also directly influence brain function, offering another neuroprotective effect and another type of indirect treatment for ND, due to the consumption of polyphenols in the diet [13,54,55,56,90]. Furthermore, since the brain–gut axis is an integral communication system of nervous, hormonal, and immune signals between the brain–microbiota–intestinal tract, it can affect the pathogenesis of diseases related to the nervous system [23].

In the case of tannins, such as Ellagitannins, found in different foods, the composition of intestinal microbiota modulates the positive effects on many pathologies, including ND and cardiovascular diseases, and cancer. Urolithins, their gut microbiota-derived metabolites, could modulate signaling pathways involved in inflammation and aging, contribute to the intestinal wall integrity, act on the brain–gut axis, induce antioxidant and anti-inflammatory responses through scavenging free radicals and ROS, upregulate HO-1 expression, or inhibit the NF-κB and MAPK or COX-2 and 5-LOX proinflammatory signaling pathway [91].

Other mechanisms through which polyphenols may be neuroprotective include their positive role in cerebrovascular and peripheral blood flow, which ultimately affects synaptic plasticity processes and cognitive function [21].

## 5. Conclusions

The continuous increase in life expectancy is inversely correlated to the quality of life during aging. NDs are accompanied by increased oxidative stress, inflammation, metal accumulation, and mitochondrial dysfunction, which are processes that can induce cell death. Several physiological mechanisms are altered by these pathological changes, contributing to the etiology of ND diseases, such as stroke, MS, PD, AD, and HD, which increase and become more prevalent with advancing age, thereby becoming a great challenge for modern societies.

With such complex mechanisms, the prevention and treatment of these disorders call for new therapeutic strategies that target multiple genes and proteins. For years, it has been known that food and health are interrelated. Polyphenols are naturally occurring secondary plant metabolites with remarkable multipotent abilities to control and modulate ROS, metal toxicity, proteins, enzymes, receptors, inflammation, apoptosis, signal transduction, ion channels, and neurotransmitters. Their direct use and dietary supplementation could act as an antioxidant and neuroprotective therapy for treating NDs. In addition, natural products, including polyphenols, represent a significant source of new leading compounds in drug discovery and development.

Most experimental and epidemiological studies suggest that dietary polyphenols activate antioxidant pathways such as Nrf2/HO1 and downregulate NFκB, MMP, PPAR, HIF-1, and STAT pathways, as well as neurotrophic factors and their pathways, such as BDNF, NGF, NT3, and NT4. In addition, polyphenols also modulate the immune response by inhibiting proinflammatory biomarkers such as CCL17/22, CCR1/2, MIP1 α/1 β, CXCL (9, 10, 11), IFN-γ, TNF-α, and IL(1 β, 6, 17A, 22), helping to reduce the characteristics of neurodegeneration, that is, oxidative damage (including chelation), inflammation, the modulation of signaling pathways related to neuronal survival and differentiation, the inhibition of neuropathological processes, the regulation of neuronal function mitochondrial, and apoptosis.

Therefore, understanding the mechanisms through which polyphenols act at the molecular level, as well as trying to improve or understand how to achieve their better bioavailability of these, their interaction with the intestinal microbiota, and their related effects is critical in proposing their use as dietary supplements to prevent neurodegenerative disorders, among some other diseases.

In conclusion, polyphenols and their metabolites are essential compounds with multiple biological activities. Their efficacy as antioxidants and their ability to modulate pro-survival and antiapoptotic signaling pathways and potentiate multiple pathways and neurotrophic factors are essential to prevent and slow down neurodegenerative disorders. In addition, since they are safe and have very low toxicity, coupled with the fact that foods or products of natural origin are readily available, it is sensible to test their efficacy in preclinical and clinical studies for the treatment and amelioration of NDs, both for isolated polyphenols and their combinations. Finally, in recent years, different polyphenols, or their synthetic derivatives have been patented as drugs against various human diseases.

## Data Availability

Not applicable.

## Sample Availability

Samples of the compounds are not available from the authors.
